# 

**Published:** 1852-09

**Authors:** 


### CONTENTS OF NO. III.

### ORIGINAL COMMUNICATIONS.

ESSAYS AND CASES.

Art. I.—Observations on Excision of the Superior Maxillary Bone; illustrated by seven Cases. By S. D. Gross, M. D., Professor of Surgery in the Medical Department of the University of Louisville. - - - - - - - 185

REVIEW.

Art. II.—A Treatise on the Practice of Medicine. By George B. Wood, M. D., Professor of the Theory and Practice of Medicine in the University of Pennsylvania; President of the College of Physicians of Philadelphia, &c. &c. Third Edition. - - - - - - - 229

BIBLIOGRAPHICAL NOTICES.

Art. III.—An Essay on the Yellow Fever, as it has occurred in Charleston, including its origin and progress up to the present time. By Thos. Y. Simons, M. D., Port Physician, formerly President of the Medical Society, and Professor of Practice of Physic in the late Medical College of South Carolina, medical adviser and formerly Chairman of the Board of Health, etc. Read before the South Carolina Association, at its Anniversary Meeting, May, 1851. - - - - - - - - - - 238

Art. IV.—On the Surgical Treatment of Polypi of the Larynx, and Oedema of the Glottis. By Horace Green, A. M., M. D., President of the Faculty, and Professor of the Theory and Practice of Medicine, in the New York Medical College; Member of the American Medical Association; Fellow of the College of Physicians and Surgeons, N. Y.; and Honorary Member of the Philadelphia Medical Society. - - - - - - - - 239

Art. V.—Illustrated Manual of Operative Surgery and Surgical Anatomy. By C. Bernard & C. Huette; edited, with notes and additions, by W. H. Van Buren, M. D., Surgeon to Bellevue Hospital; and C. E. Isaacs, M. D., Demonstrator of Anatomy in the College of Physicians and Surgeons, New York. - - - - - 242

Art. VI.—The Principles and Practice of Surgery. Illustrated by three hundred and sixteen engravings on wood. By William Pirrie, F. R. S. E., Regius Professor of Surgery in the Medical College and University of Aberdeen; Surgeon to the Royal Infirmary, etc., etc. Edited with additions by John Neill, M. D., Surgeon to the Pennsylvania Hospital, Demonstrator of Anatomy in the University of Pennsylvania, etc.

Lectures on the Principles and Practice of Surgery. By Bransby B. Cooper, F. R. S., Senior Surgeon to Guy's Hospital, etc.
The Principles of Surgery. By James Miller, F. R. S. E., F. R C. S. E., Author of a Treatise on the Practice of Surgery; Surgeon in Ordinary to the Queen for Scotland; Surgeon in Ordinary to his Royal Highness Prince Albert, for Scotland; Professor of Surgery in the University of Edinburgh; Senior Surgeon to the Royal Infirmary, etc., etc. Third American from the Second and Enlarged Edinburgh Edition. Illustrated by two hundred and forty engravings on wood. Revised, with additions by F. W. Sargent, M. D., Member of the College of Physicians of Philadelphia; Author of "Minor Surgery," etc. - - - - - - - - 243

SELECTIONS FROM FOREIGN AND HOME JOURNALS.

London Hospital.—Abscess of the Lung following Pneumonia; Death; Autopsy. . - - - - - - 245
Phlegmasia Dolens Occurring in a Female, and not Connected with the Puerperal State. - - - - - - 248
Summer Epidemics. - - - - - - - - - 249
An Eye Destroyed by a Bird-Shot. - - - - - - 250
Anesthetic Agents in Operations for Gunshot Wounds. - . 251
Chloride of Sodium in Intermittent Fever. - - - - 252
On the Bite of the Rattle Snake. - - - - - 255
Chloroform in Obstruction of the Bowels from Spasms. - - 258
Nitric Acid in Hooping-Cough and Asthma. - - - - 259
Lemon Juice in Acute Rheumatism, - - - - - 260
Fracture of the Os Brachii at the insertion of the Deltoid Muscle in a Colored Preacher while in the act of Gesticulating. 261
Croup.—Compound Tincture of Chloroform as a Remedy. - 262
Operation of Tracheotomy in an Epileptic. . - - - - 263
New Mode of Reducing Strangulated Hernia. - - - 267
Starch in Cutaneous Diseases. - - - - - - - 268

ORIGINAL INTELLIGENCE.

University of Louisville, - - - - - - - - 269
Pure Medicines, - - - - - - - - - 269
Health of Louisville, - - - - - - - - 271
Sulphur Water in Stone in the Bladder, - - - - - 272
Flint on Continued Fever, - - - - - - 273
Kentucky Medical Society, - - - - - - - 276

